# One case of condyloma acuminate

**DOI:** 10.11604/pamj.2024.47.177.42971

**Published:** 2024-04-09

**Authors:** Lin-na Lv, Zhuang-li Tang

**Affiliations:** 1 Linping Campus, The Second Affiliated Hospital of Zhejiang University School of Medicine, Hangzhou, China

## Image in medicine

A 16-year-old man presented to the dermatological department with a chronic history of cauliflower-like mass on the penis. He recalled that he first found several mung bean-sized papules on the glans without ulcers or bleeding but adopted no treatments. Thereafter the papules were gradually spread to the prepuce and the sizes of the initial lesions were enlarged dramatically. He complained of occasional itch and tingling on the glans but denied urethral discharge. On physical examination, the vital signs were within the normal ranges. The expanding and reddish papules and plaque were loosely attached to the glans and prepuce but the shaft of the penis was spared. Weighting pros and cons and after fully informed consent, we started with fractional surgery to remove the majority and the pathological examination confirmed the diagnosis. Fulguration was utilized on the remaining mass and thereafter 10 sessions of hyperthermia treatment were proposed. Punctually, the lesion completely diminished. No recurrence was detected within 6 months of follow-ups.

**Figure 1:**
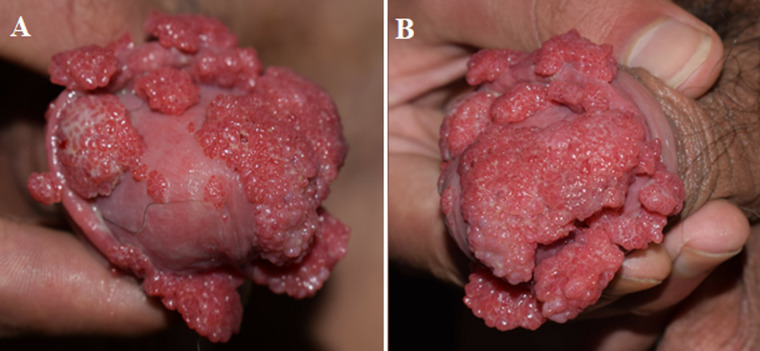
A, B) condyloma acuminata in a young Chinese man

